# Glycogen synthase kinase 3β: a key player in progressive chronic kidney disease

**DOI:** 10.1042/CS20245219

**Published:** 2025-06-17

**Authors:** Mingzhuo Zhang, Marc Tatar, Rujun Gong

**Affiliations:** 1 Division of Nephrology, Department of Medicine, University of Toledo College of Medicine, Toledo, Ohio, U.S.A.; 2 Department of Ecology, Evolution and Organismal Biology, Brown University, Providence, RI, U.S.A.

**Keywords:** GSK3, podocytopathy, CKD, Lithium, Oxidative stress

## Abstract

Chronic kidney disease (CKD) is a serious medical condition that poses substantial burdens on patients, families, healthcare systems, and society as a whole. It is characterized by progressive kidney damage and loss of function in the kidney, often compounded by underlying conditions such as diabetes, hypertension, and autoimmune diseases. Glycogen synthase kinase 3 beta (GSK3β), a highly conserved serine/threonine kinase originally implicated in insulin signaling, has emerged as a convergent point of multiple pathways implicated in the pathogenesis and progression of CKD. In the kidney, GSK3β regulates cell fate across diverse cells, including podocytes, mesangial cells, and renal tubular cells, through its interactions with key signaling pathways such as Wnt/β-catenin, NF-κB, Nrf2, PI3K/Akt, and cytoskeleton remodeling pathways. Evidence suggests that dysregulation of GSK3β is closely associated with pathological changes in the kidney, including podocyte injury, mesangial expansion, interstitial fibrosis, and tubular atrophy, which collectively drive chronic kidney destruction. In CKD, GSK3β is overexpressed and thus hyperactive in kidney cells. This sustained hyperactivity perpetuates oxidative stress and profibrotic signaling, particularly in renal tubular cells, thus accelerating the transition from acute kidney injury to CKD. Pharmacological targeting of GSK3β with selective inhibitors has shown promise in preclinical models, by reducing kidney injury, attenuating renal fibrosis, and promoting renal recovery, positioning GSK3β as a potential therapeutic target for CKD. This review highlights recent advances in understanding the molecular and cellular mechanisms through which GSK3β contributes to CKD and underscores its potential as a therapeutic target for various chronic renal diseases.

## Introduction

Chronic kidney disease (CKD) is characterized by the gradual but persistent decline of renal function, ultimately resulting in end-stage renal disease that necessitates dialysis or kidney transplantation [1,2]. CKD represents a significant global health challenge [3–5], with its prevalence steadily increasing due to aging populations, rising rates of comorbidities like diabetes, hypertension, and other risk factors. According to the Global Burden of Disease study, it is estimated that over 850 million people worldwide are affected by some form of kidney disease. CKD commonly arises from underlying conditions such as diabetes mellitus, hypertension, and autoimmune diseases, with these etiologic factors driving disease progression through common pathomechanisms such as chronic inflammation, oxidative stress, and fibrosis [6,7]. Current treatments aim to manage these underlying causes, control blood pressure, and slow kidney damage, primarily through the use of renin–angiotensin–aldosterone system inhibitors, sodium-glucose cotransporter 2 inhibitors, and other renoprotective agents [8–12]. While these therapies can delay disease progression, they often fail to halt or reverse kidney damage in many patients. Moreover, patients with advanced CKD face a higher risk of cardiovascular complications and mortality, further emphasizing the limitations of existing treatment options [13,14]. In light of these challenges, there is an urgent need for novel therapeutic strategies that more effectively address the complex and multifactorial pathophysiology of CKD.

The insulin signaling pathway plays a crucial role in kidney health and diseases, including CKD [15,16]. Within this pathway, glycogen synthase kinase 3 (GSK3) has garnered attention for its potential involvement in kidney injury and repair. Originally characterized in the early 1980 s as a key transducer of insulin signaling responsible for regulating glycogen synthesis, GSK3 is a highly evolutionarily conserved intracellular serine/threonine kinase. It functions by phosphorylating and inhibiting glycogen synthase, thereby playing a central role in glucose metabolism [17]. Beyond its metabolic function, GSK3 has been recognized as a key regulator of various cellular processes, such as proliferation, differentiation, apoptosis, and inflammation. Dysregulation of GSK3 activity has been implicated in a number of diseases, such as neurodegenerative disorders, cancer, diabetes, and inflammatory conditions, positioning it as a promising therapeutic target [18].

Mammalian GSK3 exists in two isoforms: GSK3α (51 kDa) and GSK3β (47 kDa), each encoded by distinct genes located on chromosomes 19 and 3, respectively. Although sharing 97% homology in their catalytic domains and 84% homology in overall structure, GSK3α and GSK3β are not functionally identical and redundant [17,19–21]. The fact that GSK3α knockout (KO) mice display increased insulin sensitivity and lower lipid levels, while embryos lacking GSK3β are not viable, underscores that the α and β isoforms of GSK3 are not functionally interchangeable in some critical celluar processes, with neither able to compensate for the loss of the other [22]. The two isoforms are differentially expressed in a tissue-dependent pattern. In the kidney, both GSK3 isoforms are expressed in renal parenchyma, but the β isoform rather than the α isoform of GSK3 is predominantly expressed and particularly enriched in podocytes and renal tubular cells [23]. Given these findings, most research studies focus on the role of GSK3β in the progression of kidney diseases. In contrast to the findings in global KO mice, podocyte-specific KO of only GSK3 β or GSK3α in mice either at the embryonic stage or during young adulthood resulted in no discernible phenotype, whereas combined KO of both GSK3α and GSK3β specifically in glomerular podocytes in embryonic or adult mice caused severe podocyte injury, glomerulosclerosis, heavy proteinuria, and significant impairment of kidney function [24].

Burgeoning evidence suggests that GSK3β plays a key role in the pathogenesis of CKD [25–28]. GSK3β dysregulation has been associated with various CKD, including glomerular disease, diabetic kidney disease (DKD), and chronic renal tubulointerstitial disease, in both human patients and preclinical models. In cell signaling networks, GSK3β resides at the nexus of multiple pathways involved in CKD, including mitochondria permeability transition (MPT), cytoskeletal organization, development control, insulin signaling pathway, canonical wingless (Wnt) pathway, NF-κB pathway, and the NF-E2-related factor 2 (Nrf2) antioxidant response signaling. GSK3β has emerged as the integration point of multiple kidney injury pathways and has become one of the most attractive therapeutic targets for CKD, which involves multiple pathogenic signaling mechanisms. Indeed, targeting GSK3β via genetic KO or pharmacological inhibitors can mitigate kidney injury in CKD by reducing cell death, inflammation, and fibrosis, thereby improving outcomes in preclinical models [25–27].

In light of the central role of GSK3β in renal injury and its therapeutic potential, this review consolidates current research on the involvement of GSK3β in CKD, with an aim to delineate the molecular mechanisms through which GSK3β influences CKD progression and highlight the therapeutic implications of targeting this kinase. By synthesizing insights from recent studies, this article seeks to provide a comprehensive understanding of GSK3β as a critical factor in CKD development, paving the way for innovative therapeutic strategies to enhance kidney health and improve patient outcomes.

## Molecular biology of GSK3β

### Molecular structure of GSK3β

**Figure 1:**
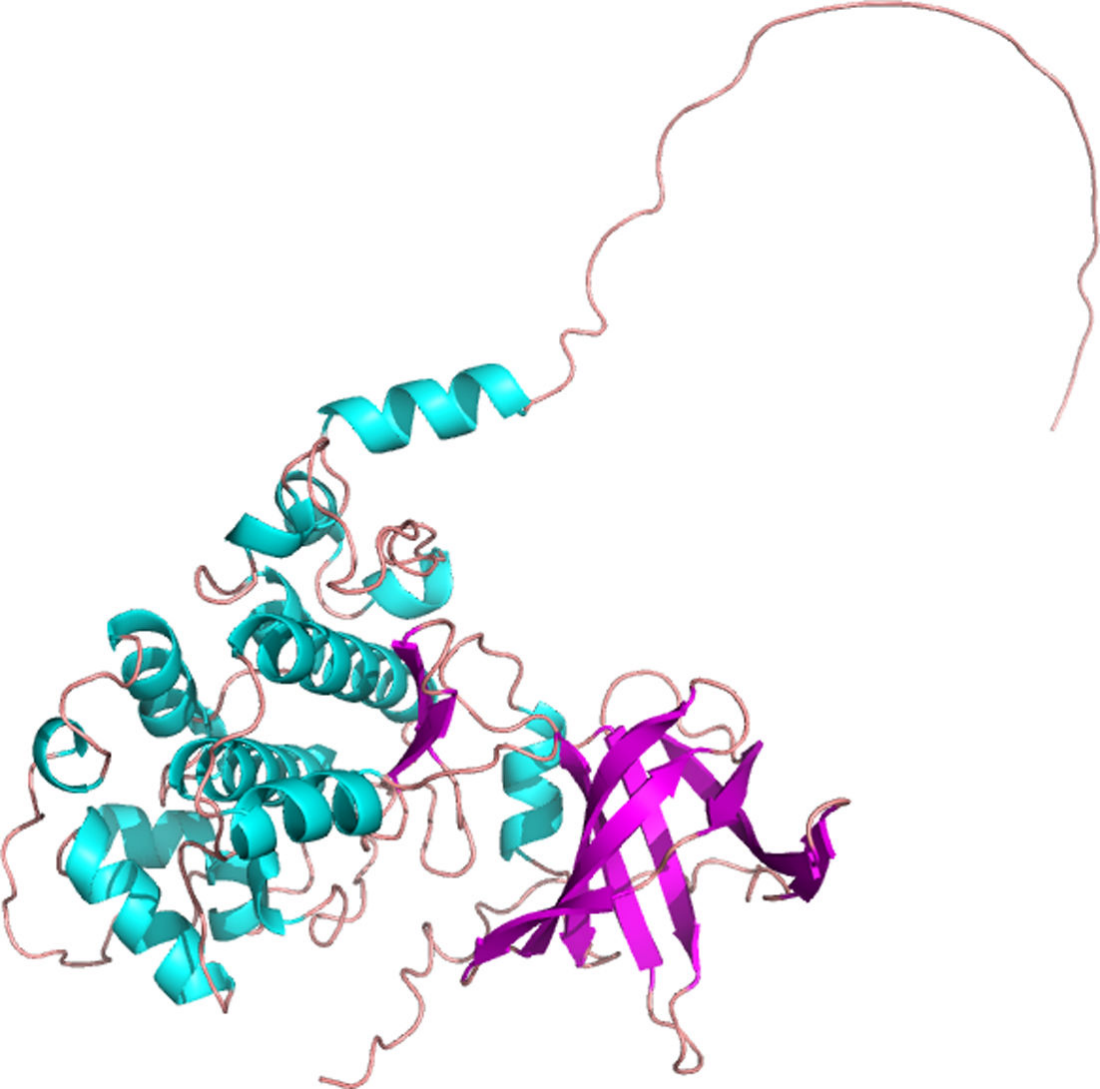
Protein three-dimensional structure modeling of GSK3β. The full-length three-dimensional protein structure of GSK3β was based on and SWISS-MODLE (https://swissmodel.expasy.org/interactive). Views of the three- dimensional model of human GSK3β.

**Figure 2:**
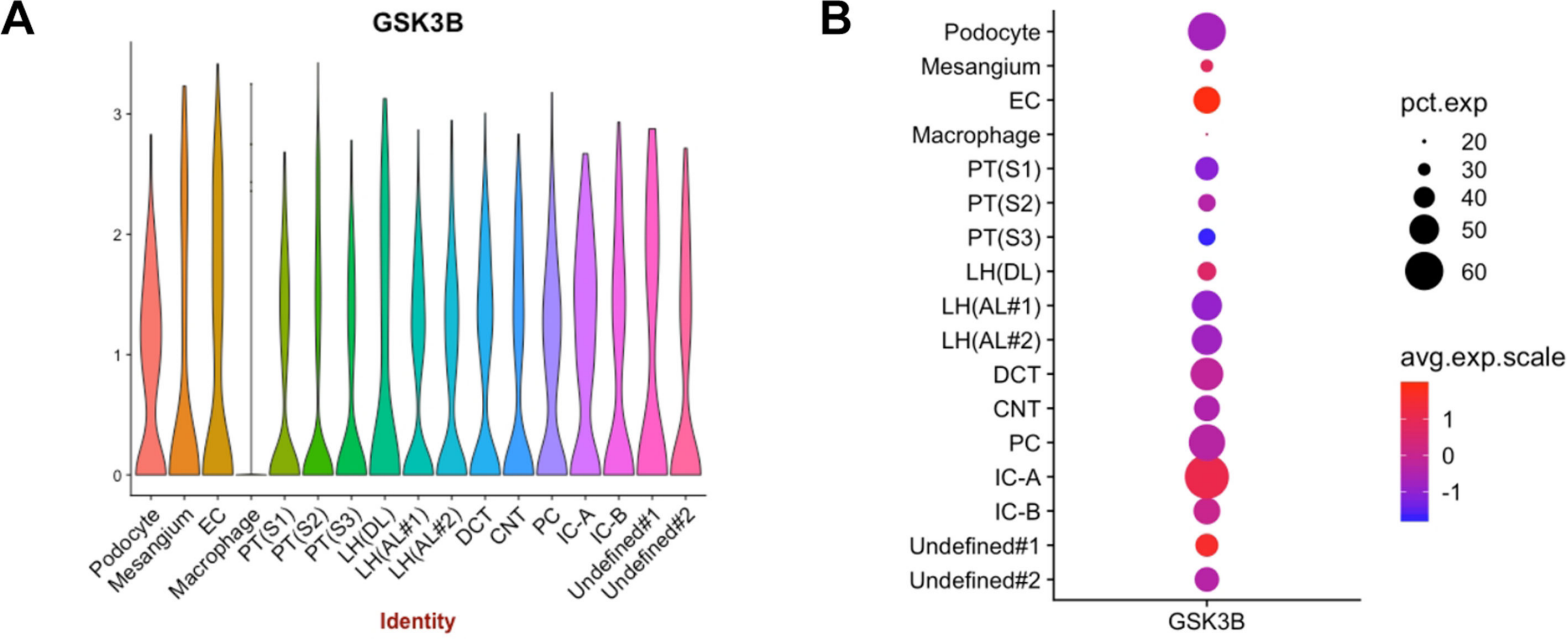
Single-cell database analysis reveals widespread expression of GSK3β across kidney cell populations. GSK3β is predominantly expressed in various kidney cell types, including podocytes, mesangial cells, and renal tubular epithelial cells. A post hoc analysis was performed on the single nucleus RNA sequencing (snRNAseq) transcriptome of human kidneys based on the Wu and Uchimura et al. Healthy Adult Kidney Dataset (RBK RID: 14–4KPM) that is publicly available from Kidney Interactive Transcriptomics (https://humphreyslab.com/SingleCell/) reveals the mRNA expression of *GSK3B* in various kidney cells (A, B) . CNT, connecting tubule; DCT, distal convoluted tubule; EC, endothelial cell; IC-A, alpha intercalated cell; IC-B, beta intercalated cell; LH (AL), loop of henle (ascending limb); LH (DL), loop of henle (descending limb); PC, collecting duct principal cell; PT (**S1**), proximal tubular (Segment 1); PT (**S2**), proximal tubular (Segment 2); PT (**S3**), proximal tubular (Segment 3)

**Figure 3:**
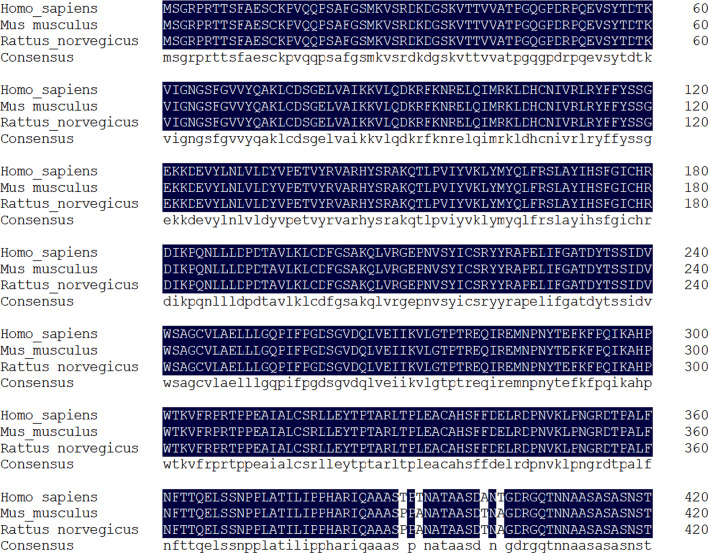
GSK3β is highly conserved in various organisms. Amino acid sequence alignment analysis of GSK3β in various organisms. Multiple sequence alignments were analyzed using the DNAMAN analysis software package (DNAMAN version 6.0).

GSK3β, encoded by the *GSK3B* gene, is a protein composed of 420 amino acids, structured with 11 exons. It features an N-terminal catalytic domain and a C-terminal regulatory domain (Figure 1) [29]. GSK3β is prominently expressed in several tissues, such as skeletal muscle [30], liver [31], adipose [32], brain [33], cardiac tissue [34], and kidney (Figure 2) [35]. Key structural elements of GSK3β include the glycine-rich loop for ATP stabilization, the catalytic loop essential for phosphoryl-transfer, and the activation loop that regulates access to the catalytic site. The catalytic domain includes the ATP-binding pocket, which is vital for its kinase activity, allowing ATP to bind and facilitate the transfer of phosphate groups to substrate proteins [36,37]. Comparative sequence analysis of GSK3β across different species, such as humans (UniProt ID: P49841), mice (UniProt ID: Q9WV60), and rats (UniProt ID: P18266), reveals a high degree of conservation in key functional regions (Table 1 and Figure 3). The wide distribution and sequence conservation of GSK3β suggest that this protein performs functions of fundamental biological importance.

**Table 1:** Protein sequence and homology analysis by BLAST for GSK3β in various organisms.

Species	Accession	GMQE	Coverage	Seq identity
Homo sapiens	NP_001139628.1	0.88	100%	100%
Mus musculus	NP_062801.1	0.88	100%	99.05%
Rattus norvegicus	NP_114469.1	0.88	100%	99.05%

GMQE, global model quality estimate.

**Figure 4:**
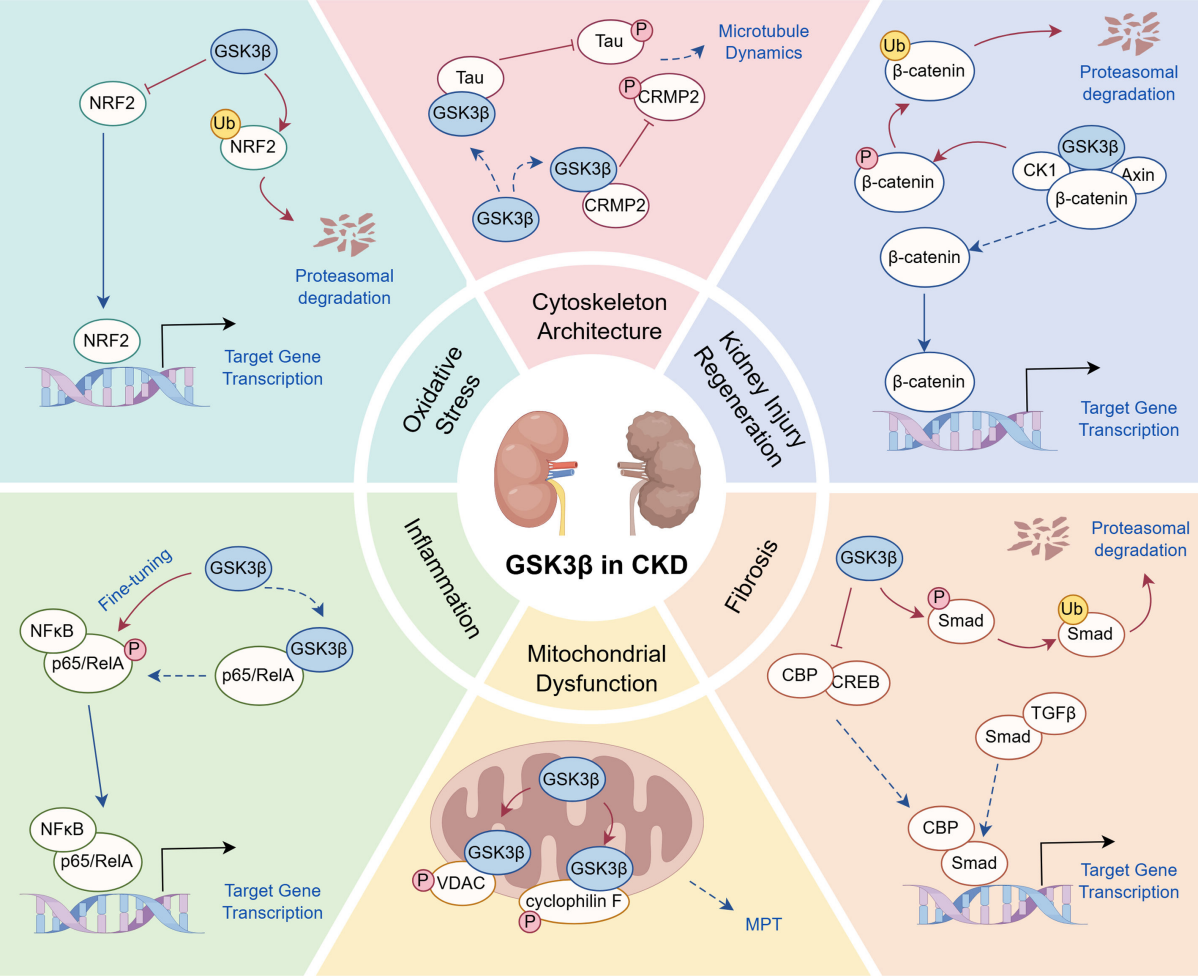
Schematic of GSK3β signaling pathways. Following activation, GSK3β triggers a variety of signaling pathways, including Wnt/β-catenin pathway, NRF2 pathway, NF-κB pathway, and TGF-β/Smad pathway, which are likely implicated in the regulation of cellular function and homeostasis by GSK3β. Beyond these pathways, GSK3β plays an important role in cytoskeletal remodeling and mitochondrial regulation, impacting cellular motility, structural integrity and viability. AKI, acute kidney injury; CBP, CREB-binding proteins; CK1, casein kinase 1; CREB, cAMP response element-binding protein; CRMP2, collapsin response mediator protein 2; MPT, mitochondria permeability transition; NRF2, NF-E2-related factor 2; P, phosphorylation; Ub, ubiquitin; VDAC, voltage-dependent anion channel. The diagram was drawn by Figdraw.

While GSK3β was initially identified as a cytoplasmic protein, recent studies have demonstrated its presence in other cellular compartments, including the nucleus and mitochondria [38]. Mitochondrial GSK3β plays a crucial role in regulating endoplasm-mitochondria calcium crosstalk [39], oxidative stress [40], and mitochondrial electron transport chain [41,42]. Even in the cytoplasm, GSK3β is not uniformly distributed but is compartmentalized in subcellular structures, such as growth cones and multivesicular bodies, suggesting that specific pools of GSK3β are involved in distinct cellular processes or signaling events. Indeed, inhibition of GSK3β through the insulin signaling pathway has a negligible effect on GSK3β-regulated Wnt/β-catenin signaling.

### Biological function of GSK3β

The activity of GSK3β is tightly regulated by specific post-translational modifications. In general, the functions of GSK3β are dependent on tyrosine residue phosphorylation (Tyr216) as an active form. Conversely, serine residue phosphorylation (Ser9) is associated with an inhibitory status [43,44]. Ubiquitination at Lysine 183 leads to a reduction in GSK3β levels by promoting its degradation through the ubiquitin-proteasome pathway, while acetylation at Lysine 15 stabilizes GSK3β by modulating its functional activity [45–49].

As a kinase, GSK3β plays a crucial role in regulating numerous biological processes [18], either by catalyzing the phosphorylation of signaling proteins that serve as GSK3β substrates or by mediating the degradation of target proteins. To date, over 100 proteins, ranging from signaling transducers and transcription factors to cytoskeleton regulators, have been identified as substrates of GSK3β, undergoing phosphorylation and regulation by its activity. Dysregulation of GSK3β-mediated signaling and cellular processes contributes to the onset and progression of numerous diseases. In the kidney, GSK3β is highly expressed in renal parenchymal cells [23], and its deficiency in the mature kidney is associated with alterations in CKD [25–28]. These observations underscore the vital role of GSK3β in maintaining kidney integrity and overall physiological stability.

## GSK3β in cellular signaling transduction

GSK3β acts as a central convergence point for multiple key cellular signaling pathways, orchestrating a range of critical processes that regulate cellular function and homeostasis. It plays a pivotal role in the Wnt/β-catenin pathway, which is crucial for cell proliferation, differentiation, development, as well as tissue injury, repair, and regeneration. GSK3β is also a pivotal modulator of the NRF2 pathway, which controls oxidative stress responses and cellular defense mechanisms. Moreover, GSK3β is integral to the TGF-β (the transforming growth factor-beta)/Smad3 signaling pathway, where it influences cellular differentiation and fibrogenesis. In addition, GSK3β interacts with the phosphatidylinositol 3-kinase (PI3K)/AKT pathway, a key mediator of insulin signaling and cellular survival. GSK3β is involved in the NF-κB pathway, which is central to the inflammatory reaction and immune response. Beyond these pathways, GSK3β plays an important role in cytoskeletal remodeling and mitochondrial regulation, impacting cellular motility, structural integrity and viability. Through its broad involvement in these essential cellular processes, GSK3β emerges as a critical regulator of both normal cellular processes and pathogenesis of various diseases, including CKD (Figure 4).

### GSK3β and Wnt/β-catenin signaling pathway

GSK3β functions as a negative regulator in the classical Wnt/β-catenin pathway by promoting the degradation of β-catenin [29]. In the absence of Wnt activation signals, the Wnt-specific scaffold protein Axin forms a complex with GSK3β, β-catenin, and casein kinase I. This complex facilitates the phosphorylation of β-catenin at Ser45 by casein kinase I, followed by phosphorylation at Thr41, Ser33, and Ser37 by GSK3β, leading to β-catenin ubiquitination and subsequent degradation. In contrast, when Wnt activation signals are present, Wnt activates Dvl, which, in conjunction with the GSK3β-binding protein Frat, facilitates the dissociation of the Axin complex [29,50–53]. This dissociation prevents β-catenin phosphorylation, allowing its stabilization and activation. Cross-talk between GSK3β and the Wnt/β-catenin pathway has been implicated in the development of renal fibrosis [54,55]. The role of GSK3β in the Wnt/β-catenin signaling pathway during kidney injury, however, remains controversial. Moderate activation of the Wnt/β-catenin pathway supports kidney cell survival and regeneration, promoting recovery and minimizing initial damage. Conversely, excessive Wnt/β-catenin activation can drive fibrosis by increasing extracellular matrix production, activating fibroblasts, exacerbating inflammation through up-regulation of cytokines and chemokines, resulting in podocyte injury along with interstitial fibrosis and tubular atrophy. Some studies have suggested that the ubiquitination and subsequent degradation of GSK3β may activate Wnt/β-catenin signaling, which in turn contributes to the progression of acute kidney injury (AKI) [56]. However, conditional ablation of GSK3β in podocytes had minimal effect on the β-catenin pathway and did not result in renal phenotypes in young mice, as demonstrated in our study [28] and supported by findings from Hurcombe et al. [24]. This lack of impact is likely due to compensation by GSK3α, which seems to function redundantly with GSK3β in mediating Wnt/β-catenin signaling [57].

### GSK3β and NF-κB signaling pathway

The activation of NF-κB is a tightly regulated process initiated by upstream signaling cascades that activate the IκB kinase complex. Under basal conditions, NF-κB dimers, predominantly the p65/p50 heterodimer, are retained in the cytoplasm through interaction with the inhibitory protein IκB [22,58]. Upon stimulation, IκB kinase complex phosphorylates IκB, triggering its ubiquitination and subsequent proteasomal degradation. This process exposes a nuclear localization sequence on the NF-κB dimer, facilitating its rapid translocation into the nucleus, where it binds to specific DNA sequences and initiates the transcription of genes involved in inflammation [59]. GSK3β has been shown to enhance NF-κB transcriptional activity by phosphorylating key components, such as the p65 (RelA) subunit. More specifically, the C-terminal region of RelA/p65 contains a highly conserved domain that shares homology with the consensus docking sequence for GSK3β, which is essential for the efficient phosphorylation of Ser-468 and facilitating the physical interaction between RelA/p65 and GSK3β [60]. Additionally, GSK3β regulates NF-κB activation and RelB degradation through site-specific phosphorylation of BCL10, and it also influences histone modifications, which further modulate the expression of inflammatory genes by facilitating the binding of NF-κB and the p65/p50 complex to promoter regions [61–63]. GSK3β also interacts with PP2Ac to regulate NF-κB activity, and the novel GSK3β inhibitor 5*n* has been found to protect against AKI through PP2Ac-dependent mechanisms [64]. Furthermore, GSK3β inhibitors reduce oxidative stress and production of inflammatory mediators by inhibiting NF-κB p65 and IκB phosphorylation via the TLR/MyD88 pathway [65]. Mirabegron, through modulation of the GSK3β/NF-κB p65 signaling pathway, exerts protective effects on renal tissue and preserves renal function in the context of renal ischemia reperfusion [66]. Thus, GSK3β plays a crucial role in regulating inflammation via the NF-κB pathway, significantly contributing to renal inflammation and chronic kidney disease [67,68]. Targeting GSK3β therefore represents a promising therapeutic strategy for treating inflammation in CKD.

### GSK3β and NRF2 signaling pathway

As the master regulator of antioxidant responses, Nrf2 is subjected to a complex and meticulously regulated pathophysiological process that is governed by various signaling pathways [26,69]. Under conditions of low oxidative stress, Nrf2 is retained in the cytoplasm, where it is bound to the inhibitory protein Kelch-like enoyl-CoA hydratase-associated protein 1 (Keap1). However, upon oxidative stress, Nrf2 is activated, following its dissociation from Keap1 and subsequent translocation into the nucleus. In the nucleus, Nrf2 binds to a conserved antioxidant response element (ARE), initiating the transcription of cytoprotective genes [70]. Following severe and persistent kidney injury, the sustained hyperactivity of GSK3β impairs the nuclear accumulation of Nrf2 through a Keap1-independent mechanism, exacerbating oxidative stress and playing a pivotal role in the progression from AKI to CKD [26]. Inhibition of GSK3β, for example by low-dose lithium, may exert protective effects through direct regulation of the Nrf2 antioxidant response [71]. In typical circumstances, short-term and mild kidney injury would activate the reperfusion injury salvage kinase (RISK) pathway, which is a major prosurvival signaling cascade that embraces the PI3K/Akt pathway and its downstream target, GSK3β [72]. Activation of the RISK pathway inhibits GSK3β, thereby preventing the degradation of NRF2, allowing NRF2 to accumulate, translocate to the nucleus, and activate the expression of antioxidant genes. This underscores the pivotal role of GSK3β in regulating NRF2 stability and function in CKD [73].

### GSK3β and TGF-β/Smad signaling pathway

The TGF-β signaling pathway is a crucial regulator of various cellular processes, including proliferation, differentiation, apoptosis, and fibrogenesis [74–76]. The pathway is initiated when TGF-β ligands, such as TGFβ1, TGFβ2, and TGFβ3 (77–79), bind to TGF-β receptors [80,81], leading to the phosphorylation of receptor-regulated Smad proteins, mainly Smad2 and Smad3 [82,83]. These phosphorylated Smads then form a complex with Smad4, which translocates to the nucleus to regulate the transcription of specific target genes [84,85]. GSK3β is a key regulator of the TGF-β1 pathway [78]. In the heart, diminished GSK3β activity enhances TGF-β1-induced phosphorylation of Smad3 at the C-terminal Ser423/425 site (which leads to activation), indicating that GSK3β typically inhibits this phosphorylation [86]. Additionally, GSK3β controls Smad3 stability by phosphorylating it at the Thr66 site, leading to its ubiquitination and degradation [87].

The Smad linker region, situated between the MH1 and MH2 domains, plays a distinct role in Smad function compared to the C-terminal domain [88]. While phosphorylation of the C-terminal domain activates Smad, phosphorylation within the linker region inhibits its transcriptional activity [89]. It is proposed that GSK3β-mediated phosphorylation at Ser204 in the linker region provides an additional mechanism to suppress Smad3 activity [90]. Furthermore, GSK3β inhibition enhances the activity of cAMP response element-binding protein (CREB), which competes for CREB-binding protein (CBP) — a transcriptional coactivator essential for TGF-β1/Smad signaling to drive the profibrogenic plasticity in tubular epithelial cells (TECs) [91]. GSK3β also indirectly influences TGF-β/Smad signaling through cross-regulation with other signaling pathways, including Wnt/β-catenin signaling [29,92–94].

### GSK3β and PI3K/Akt signaling pathway

The PI3K/Akt signaling is the core of the prosurvival RISK pathway that is triggered by insulin or a variety of growth factors. It represents a critical transduction cascade that is essential for orchestrating a range of essential cellular processes, including proliferation, survival, metabolism, migration, and apoptosis [95,96]. Activation of PI3K results in the production of phosphatidylinositol [3,4,5]-triphosphate, which activates Akt. Subsequently, Akt phosphorylates a diverse range of target proteins, thereby regulating a multitude of cellular functions [95,96]. A key target of the PI3K/Akt pathway is GSK3β [97]. Under basal conditions, GSK3β is active, regulating glycogen biosynthesis, cell cycle progression, and apoptosis. However, upon Akt activation, GSK3β is phosphorylated at the Ser9 residue, resulting in its inactivation [98]. This inactivation is particularly significant in processes such as TGF-β1-induced epithelial-mesenchymal transition (EMT) [98]. Akt phosphorylates and inactivates GSK3β, leading to the stabilization of proteins such as cyclin D1, indicating the role of the PI3K/Akt/GSK3β signaling pathway in regulating cell survival and proliferation [99]. Notably, while GSK3β is generally inactivated by the PI3K/Akt pathway upon insulin treatment, exposure of kidney cells to a type 2 diabetic milieu containing high ambient insulin and glucose as well as microinflammation causes a reduction in Ser9 phosphorylation, leading to GSK3β hyperactivity. This overactivated GSK3β can then phosphorylate insulin receptor substrate 1 (IRS1) at the Ser332 site, impairing insulin signaling and contributing to insulin resistance, a condition often associated with diabetic nephropathy [100].

### GSK3β and cytoskeleton remodeling

The cytoskeleton is essential for maintaining cellular shape, internal organization, and polarity in various cell types, including kidney cells. GSK3β is a central regulator of cytoskeletal dynamics, playing a crucial role in maintaining kidney cell function and influencing cellular response to injury [101–103]. It modulates microtubule-associated proteins (MAPs) crucial for microtubule stability and organization [104] and influences the actin cytoskeleton by regulating Rho family GTPases, which are vital for cell migration and adhesion [105,106]. As highly specialized epithelial cells that cover the exterior of the glomerular capillary and regulate glomerular permeability, podocytes possess an enriched actin and microtubule cytoskeletal network. Proper balance in cytoskeletal dynamics is critically required for podocytes to maintain the integrity of the glomerular filtration barrier. Recent research has highlighted the role of GSK3β in the dynamic remodeling of the cytoskeleton in podocytes [102]. GSK3β regulates the activity of MAPs, such as Tau and collapsin response mediator protein 2 (CRMP2), which are vital for microtubule polymerization and stability [107,108]. Upon podocyte injury induced by adriamycin, GSK3β is overactivated, and this results in the hyperphosphorylation of Tau and CRMP2, leading to microtubule destabilization, retraction of podocyte processes, and cellular dysfunction. Conversely, inhibition of GSK3β, as observed with lithium treatment, counteracts these effects by stabilizing microtubules and promoting extension and repair of podocyte processes, which are essential for glomerular adaptation to podocyte loss [102]. Dysregulation of GSK3β has been associated with CKD such as diabetic nephropathy and glomerulosclerosis, where changes in cytoskeletal dynamics exacerbate disease progression [109]. Thus, GSK3β emerges as a key integrator of cellular function and structure in kidney cells, making it a promising therapeutic target.

## Role of GSK3β in CKD

CKD is a heterogeneous condition caused by a wide range of etiologies, each with distinct underlying pathomechanisms, clinical presentations, and progression patterns, all of which led to chronic impairment of kidney function and structure. CKD is characterized by progressive decline in kidney function and gradual, persistent renal destruction, accompanied by complex pathological features, including glomerulosclerosis, interstitial fibrosis, tubular atrophy, and inflammatory infiltration, all of which contribute to disease progression [110–112]. Accumulating evidence indicates that GSK3β is aberrantly activated in injured renal parenchymal cells, where it exacerbates CKD by promoting fibrogenesis, glomerulosclerosis, and tubulointerstitial damage [25,26,91,113–115]. Mechanistically, GSK3β participates in multiple pathogenic pathways, including the Wnt/β-catenin pathway, TGF-β/Smad pathways, inflammation cascades, and apoptosis regulation. Its hyperactivity is particularly prominent in DKD, correlating with glomerular injury, tubular damage, and proteinuria [16,109,113]. In addition, inhibiting GSK3β may effectively slow the progression from AKI to CKD by mitigating renal fibrosis [25,26]. Beyond parenchymal renal cells, GSK3β also modulates immune responses across multiple immune cell populations, which also significantly influence kidney inflammation and CKD pathogenesis. In macrophages, dendritic cells, B lymphocytes, and T lymphocytes, GSK3β governs cytokine production, metabolic reprogramming, antigen presentation, and immune polarization—processes that may underpin both acute and chronic immune-mediated renal inflammation [116–118]. Based on these findings, GSK3β is considered a crucial therapeutic target in CKD.

### GSK3β in polycystic kidney disease

Polycystic kidney disease (PKD) is an inherited disorder characterized by the gradual formation and expansion of fluid-filled cysts in the kidneys. These cysts exert pressure on healthy renal tissue, and this can eventually lead to impaired kidney function [119–122]. The pathogenesis of PKD is intricate, involving dysregulated cell proliferation, abnormal fluid secretion, and aberrant extracellular matrix remodeling within the kidneys. Recent studies have highlighted the critical involvement of GSK3β in PKD pathogenesis, particularly in regulating epithelial cell differentiation, polarity, and proliferation[123]—processes that are disrupted in PKD. GSK3β expression is significantly elevated in collecting ducts in PKD, where it mediates responses to AVP, leading to increased proliferation and fluid secretion [124–126]. Collecting ducts are central to cyst formation, and GSK3β inhibition has been shown to reduce cyst formation and renal cyst hyperplasia [126,127]. Elevated renal cAMP levels are a hallmark of PKD [128,129]. Notably, GSK3β modulates AVP-sensitive adenylate cyclase activity and cAMP production. Targeted GSK3β inhibition or renal-specific GSK3β KO can markedly diminish adenylate cyclase activity and cAMP accumulation [126,130,131]. GSK3β operates within a feed-forward mechanism involving cAMP- and CREB-mediated signaling pathways. In this loop, elevated cAMP levels enhance the activity of the transcription factor CREB, which binds to the GSK3β promoter region, thereby upregulating the expression of GSK3β [127]. In turn, GSK3β positively regulates the production of cAMP. The inactivation or deletion of GSK3β suppresses adenylate cyclase activity and reduces cAMP synthesis [131]. In PKD, FoxM1 promotes cyst growth in PKD1 mutant ADPKD [132]. Conversely, in AKI, GSK3β acts as an upstream suppressor of FoxM1, with GSK3β inhibition or gene deletion leading to increased FoxM1 expression and improved tubular repair [27]. Reasons for the above conflicting results are unclear but highlight the dual role of FoxM1, warranting further investigation to elucidate the underlying mechanisms. While GSK3β’s role in later-stage PKD, particularly in cyst expansion and fibrosis, is well-documented, its involvement in the early stages of the disease remains less clear [126,131,133]. In juvenile rats, LiCl exposure induced microcysts in collecting ducts, whereas in adult rats, prolonged LiCl treatment increased cell proliferation without cyst formation [134,135]. Kjaersgaard et al. found an inverse association between renal GSK3β activity and postnatal kidney development. Specifically, GSK3β facilitates cell proliferation at early stages, whereas its elevated abundance or activity sustains an anti-proliferative effect in mature kidneys. Lithium, by acting on the distal nephron—particularly early collecting duct epithelial cells—attenuates GSK3β activity, leading to dysregulated cell proliferation associated with dilatations and microcysts and subsequent structural changes [135]. Previous research has associated chronic lithium treatment with renal microcyst formation [136], and even rarely with concomitant occurrence of PKD [137]. Autopsy findings from long-term lithium users revealed microcysts and tubular dilation in both renal cortex and medulla [138], with structural alterations most prominent in the collecting ducts and distal tubules [139]. These lithium-induced microcysts are GSK3-positive with enhanced inhibitory phosphorylation of GSK3β at serine 9, and the cyst epithelium is proliferative [140]. In human biopsy samples from lithium users, renal microcysts show AQP2 positivity, implicating the collecting duct as their likely origin [135]. β-catenin is a key target of GSK3 signaling, and overexpression of β-catenin in murine renal epithelial cells has been shown to cause severe cystic changes shortly after birth [141]. As a classic GSK3 inhibitor, lithium at high doses activates the Wnt/β-catenin pathway [142]. Thus, it is plausible that renal microcyst formation following chronic lithium treatment could, at least in part, be attributed to the overactivation of the β-catenin pathway. However, studies by Doble et al. [57] demonstrate that inactivation of at least three of the four alleles of GSK3α and GSK3β is necessary to induce significant changes in β-catenin activity. This implies that there may be a potential therapeutic window during which pharmacologic GSK3β inhibition could be achieved without altering β-catenin activities in peripheral organs such as the kidney. While long-term high-dose lithium use for treating psychiatric disorders is associated with an increased risk of renal toxicity, recent evidence indicates that short-term low-dose lithium may suppress GSK3β activity without activating β-catenin, paradoxically offering renoprotective effects.

### GSK3β in DKD

DKD is one of the most common complications of diabetes mellitus and is a principal cause of chronic kidney disease. Its prevalence is on the rise, correlating with the escalating incidence of diabetes mellitus [143,144]. As a key transducer of insulin signaling, GSK3β plays a central role in regulating glucose homeostasis by phosphorylating glycogen synthase, thereby affecting glycogen synthesis, insulin signaling, and glucose metabolism [31,145]. Dysregulation of GSK3β has been associated with insulin resistance and the development of DKD [16,109,113,146,147].

As a diagnostic criterion of DKD, albuminuria is an indicator of early impairment of the glomerular filtration barrier by diabetes. Podocytes are a crucial constituent of the glomerular filtration barrier. In the progression of DKD, they can undergo apoptosis, detachment, or structural changes like foot process effacement, cytoskeletal remodeling, and glomerular basement membrane thickening, all of which contribute to the disruption of the filtration barrier [102,148,149]. Recent experimental and clinical studies have highlighted the important role of GSK3β in the pathogenesis of DKD and podocyte injury. For example, Paeng et al. [149] demonstrated that GSK3β is overactive in podocytes exposed to high ambient glucose, contributing to podocyte apoptosis. Additionally, GSK3β hyperactivity has been observed in glomerular podocytes under type 2 diabetic conditions, associated with IRS1 hyperphosphorylation, impaired Nrf2 antioxidant response, and premature senescence, underscoring its potential as a therapeutic target in type 2 diabetic glomerular injury [43]. Sarsasapogenin has been shown to ameliorate experimental DKD by targeting the GSK3β signaling pathway. This beneficial effect is associated with restoration of podocyte autophagy [150]. Similarly, hepatocyte growth factor has been found to prevent high glucose (HG)-induced podocyte injury through an autophagy-promoting mechanism that is dependent on GSK3β inhibition [151], further implicating the PI3K/Akt-GSK3β axis in lysosomal function improvement and DKD protection [152]. TDAG51 deficiency has been identified as a protective factor against HG-induced podocyte damage, primarily through the activation of Nrf2 by modulating the AKT-GSK3β pathway, further illustrating the multifaceted role of GSK3β in diabetic podocyte injury [153]. These findings collectively underscore the potential of GSK3β as a central therapeutic target for mitigating podocyte injury and delaying the progression of DKD. A number of pharmacological agents have GSK3β inhibitory activity. For instance, β-hydroxybutyrate is able to directly target the ATP-binding pocket of GSK3β and thereby block its kinase activity, as evidenced by molecular modeling and docking analysis. Through inhibiting GSK3β, β-hydroxybutyrate reinforces Nrf2 antioxidant response in glomerular podocytes, resulting in reduced senescence and glomerular injury in mouse models of type 1 DKD [35]. It is conceivable that therapeutic targeting of GSK3β for treating DKD is feasible by using existing or novel medications with GSK3β inhibitory activities.

In addition to glomerular podocytes, glomerular mesangial cells and renal TECs are injured in DKD and are involved in the pathogenesis of DKD. A growing body of evidence suggests that GSK3β also plays a role in diabetic mesangial and tubular injuries [154,155]. DKD may present with mesangial cell proliferation and expansion of mesangial matrix, leading to the formation of typical Kimmelstiel–Wilson nodules. Lin et al. [156] demonstrated that HG activates GSK3β and caspase-3, leading to apoptosis in glomerular mesangial cells. Pharmacological inhibition of GSK3β with specific inhibitors like bromoindirubin-3'-oxime or LiCl counteracts these effects by enhancing Akt phosphorylation and β-catenin translocation. Furthermore, knockdown of GSK3β reduces proliferation of mesangial cells in the DKD [157]. Diabetic kidney injury is accompanied by renal tubulointerstitial lesions, which are known to be closely associated with the decline in renal function [158,159]. GSK3β is overactivated in renal tubular cells subjected to hyperglycemic stimulation. Inhibiting the GSK3β/β-TrCP axis enhances NRF2-ARE binding and stabilizes NRF2, which in turn alleviates renal tubular injury [160]. Phosphorylated GSK3β plays a role in the elevation of SREBP-1 expression and lipid deposition in renal tubular cells following HG treatment [161]. Consistent with these, the results of renal biopsy specimens from patients at different stages of diabetic nephropathy demonstrated a consistent increase in the expression of total and activated GSK3β in glomeruli and tubules, correlated with the severity of diabetic nephropathy [162]. Contrary to the above data suggesting the pathogenic role of GSK3β hyperactivity in DKD, Mariappan et al. found that activation of GSK3β has a beneficial effect on diabetes-induced kidney damage [163]. They administered sodium nitroprusside, a nitric oxide donor that is able to activate GSK3β, to mice with streptozotocin-induced type 1 diabetes and observed improvements in renal hypertrophy, matrix protein accumulation, and albuminuria, independent of any correction of hyperglycemia. Although nitroprusside activates GSK3β, it is not a GSK3β-selective agonist. Additionally, nitroprusside is known to exert hypotensive effects, which may confound the interpretation of the improved albuminuria and diabetic kidney injury in their study. Collectively, while most studies suggest a detrimental role of GSK3β activation in DKD, to conclusively define the role of GSK3β in DKD, it is necessary to employ DN models with kidney-specific GSK3β hyperactivity or KO, or to use highly selective and rigorous inhibitors.

### GSK3β in glomerular diseases

Glomerular diseases represent one of the leading causes of CKD [4]. The progression of glomerular disease is dependent on the damage to glomerular cells, such as glomerular podocytes. Injury to glomerular cells often results in the hallmark clinical features, including proteinuria and a decline in renal function [164]. While the exact pathomechanisms of glomerular injury are obscure, converging evidence recently points to a role for GSK3β.

In cultured glomerular podocytes, doxorubicin induced cell death and disruption of the actin cytoskeleton, accompanied by marked overactivation of GSK3β and minimal activation of Nrf2. Ectopic expression of a kinase-dead mutant of GSK3β in cultured podocytes enhanced doxorubicin-induced Nrf2 activation and mitigated podocyte injury. In contrast, expression of the constitutively active mutant of GSK3β attenuated the Nrf2 response to doxorubicin and exacerbated podocyte damage [73]. In mouse models of doxorubicin nephropathy and nephrotoxic serum nephritis, podocyte-specific KO of GSK3β or pharmacological inhibition with SB216763 (a highly selective inhibitor of GSK3) significantly ameliorated podocyte injury and reduced albuminuria. This effect was primarily dependent on the restoration of NRF2 antioxidant response [73,165]. GSK3β inhibitor (such as TDZD-8), or targeted GSK3β KO in podocytes, has been demonstrated to preserve actin cytoskeleton integrity following adriamycin insult [28,40]. These interventions decreased the phosphorylation and activation of paxillin, a focal adhesion-associated adaptor protein, and reduced podocyte hypermotility [166]. Notably, a discrete pool of GSK3β located within podocyte mitochondria interacts with and phosphorylates cyclophilin F, a critical component of the MPT. Treatment with TDZD-8 effectively inhibits this phosphorylation event, thereby preventing the onset of oxidative stress-induced mitochondrial dysfunction and offering substantial protection against podocyte death [40]. Recent studies have extended the pathogenic scope of GSK3β beyond lipopolysaccharide (LPS)- or adriamycin-induced models to other glomerular diseases relevant to human pathology. In kidney biopsies from patients with primary focal segmental glomerulosclerosis (FSGS), increased expression of phosphorylated GSK3β in podocytes was observed [167]. Moreover, glomerular expression of GSK3β was elevated by 2.6-fold in FSGS and 3.1-fold in lupus nephritis in human kidney biopsies based on computerized morphometric analysis [28]. Elevated GSK3β levels were also detected in peripheral blood mononuclear cells from patients with FSGS and membranous nephropathy [168]. Furthermore, GSK3β contributes to lupus nephritis development in lupus-prone mice, in part through activation of the NLRP3/IL-1β axis. Notably, the selective GSK3β inhibitor TDZD-8 effectively blocked NLRP3/IL-1β activation in peripheral blood mononuclear cells from patients with systemic lupus erythematosus [117].

In addition to the regulation of NRF2-mediated antioxidant response, podocyte cytoskeleton integrity and MPT, GSK3β plays a pivotal role in the canonical Wnt/β-catenin signaling pathway which has been recently implicated in primary glomerular disorders [67,68,115,142]. Balanced Wnt/β-catenin signaling in podocytes is known to be critical for glomerular filtration barrier maintenance [169]. In consistency, Dai et al*.* [142] triggered Wnt/β-catenin signaling in mice with high-dose LiCl, resulting in podocyte damage and transient proteinuria. Although these findings may align with clinical observations of lithium nephrotoxicity [170–172], it is important to highlight that lithium nephrotoxicity is uncommon in clinical practice, and its severity is influenced by both the dose and duration of lithium administration [173]. The current FDA-approved dose of lithium is high to ensure that lithium effectively crosses the blood-brain barrier for the treatment of psychiatric disorders [174,175]. In contrast, our recent study demonstrates that low-dose lithium can effectively inhibit GSK3β activity and confer protection on peripheral organs, including the kidneys, without causing adverse side effects [35,176,177]. Additionally, podocyte-specific KO of GSK3β showed minimal impact on the β-catenin pathway, and this is likely due to the functional redundancy between GSK3β and GSK3α [24,28,57]. Therefore, the use of highly selective GSK3β inhibitors or lithium at low doses presents a promising therapeutic approach for the treatment of glomerular diseases.

Progressive CKD is associated with *de novo* acquisition of pro-inflammatory phenotypes by kidney epithelial cells, like glomerular podocytes, contributing to renal inflammation and dysfunction. NF-κB signaling plays a central role in this pro-inflammatory phenotypic shift and can be modulated by GSK3β. NF-κB acts as a double-edged sword in the pathogenesis of glomerular diseases [67,68,178–180]. On one hand, NF-κB directs the transcription of a multitude of injurious mediators involved in podocyte injury, most of which are related to inflammatory response and immune reaction, including proinflammatory cytokines (e.g. MCP-1) and immune regulatory cathepsin L and B7-1, which also have been implicated in podocyte cytoskeleton disorganization [181]. On the other hand, NF-κB is a survival factor essential for cellular response to stress or injury, functioning for self-protection. The actual pathobiological effects of NF-κB are dictated by its post-translational modifications, such as phosphorylations, whose patterns may influence the recruitment of different transcriptional cofactors, direct distinct profiles of gene expression, and result in varying and even opposing actions. Broad-range inhibition of NF-κB unselectively suppresses the expression of a broad range of NF-κB target genes, mitigates podocyte acquisition of pro-inflammatory phenotypes, such as expression of MCP-1, cathepsin L, and B7-1, but unfortunately sensitizes podocytes to death. In contrast, GSK3β phosphorylates NF-κB specifically at serine 467, which specifies the expression of selective NF-κB target molecules, including podocytopathic mediators like MCP-1, cathepsin L, and B7-1, but not the pro-survival Bcl-xL [182]. Inhibition of GSK3β by lithium or TDZD-8 mitigated the expression of podocytopathic mediators, ameliorated podocyte injury, but barely affected Bcl-xL expression or sensitized apoptosis. *In vivo*, in mice with LPS or adriamycin-induced podocytopathy, GSK3β inhibitors attenuated RelA/p65 phosphorylation specifically at serine 467 and improved proteinuria and podocyte injury. In contrast, TPCK, a broad-range inhibitor of NF-κB, improved albuminuria and podocyte injury by a much lesser extent but aggravated glomerular cell apoptosis. Collectively, GSK3β-directed fine-tuning of NF-κB might serve as a novel therapeutic target for glomerular disease.

### GSK3β in renal tubulointerstitial fibrosis

Renal fibrosis is a hallmark of progressive CKD, characterized by tubular atrophy and the excessive deposition of extracellular matrix (ECM) within the tubulointerstitium. Regardless of the original etiology of kidney disease—whether ischemia-reperfusion, nephrotoxicity, or renal obstruction—the renal tubule possesses an intrinsic capacity for self-repair. However, when the extent of injury surpasses the adaptive ability of the kidney for self-repair, a cascade of maladaptive kidney repair may be triggered. These include G2/M cell cycle arrest, cellular dedifferentiation, premature senescence, and the production of numerous fibrotic and pro-inflammatory mediators [183,184].

In mouse models of acute tubular injury, the tubular activity of GSK3β was significantly increased. Inhibition of GSK3β ameliorated renal injury by ameliorating tubular cell apoptosis and injury, indicating the potential value of GSK3β in blocking the progression of AKI and the subsequent transformation to CKD [185]. Analysis of the renal transcriptome database showed that patients with progressive CKD exhibited GSK3β overexpression in the renal tubulointerstitium, where the pre-defined hallmark gene sets involved in fibrogenesis were remarkably enriched [91]. Our study demonstrated that targeted inhibition of GSK3β in renal tubules—either through genetic KO or inhibitor treatment—potentiates Nrf2 antioxidant response via a Keap1-independent mechanism to ameliorate renal oxidative stress and attenuate AKI to CKD transition [26]. Additionally, GSK3β inhibition promotes CREB activity in renal tubules in FA-elicited progressive CKD [91]. CREB competes for CBP, a transcriptional coactivator essential for the TGF-β1/Smad signaling pathway to drive the profibrogenic plasticity of renal TECs. This resulted in downregulation of the transcriptional activity of the TGFβ1/Smad pathway, leading to an amelioration of renal fibrosis. Mechanistically, it is postulated that CREB serine 129 lies in the consensus sequence motif of GSK3β, and phosphorylation by GSK3β at serine 129 may act as a suppressive signal for CREB activity [186–188]. Consistently in mouse models of progression of AKI caused by ischemia-reperfusion injury to CKD, Singh et al. [189] demonstrated that pharmacological inhibition of GSK3β using TDZD-8 significantly attenuated the development of renal fibrosis. This protective effect was primarily mediated through the suppression of the TGFβ1/Smad3 signaling pathway, which in turn reduced the deposition of extracellular matrix components such as collagen-1 and fibronectin. Moreover, constitutive activation of GSK3β was found to directly enhance α-SMA expression, further implicating GSK3β in the fibrotic process. Additional recent evidence demonstrates that inhibition of GSK3β enhances transcription factor EB -mediated lysosomal synthesis and facilitates ECM degradation, thereby improving renal fibrosis [190].

## The application of GSK3β inhibitors for treating kidney diseases

A variety of medications with GSK3β inhibitory activities have been used to treat a range of diseases, and several of these have already undergone clinical trials (Table 2, https://clinicaltrials.gov/). However, there remains a significant lack of clinical studies evaluating GSK3β inhibitors for renal diseases [191]. In fact, the majority of research on GSK3β inhibitors in the context of kidney disease is still in the preclinical phase, relying on animal models and *in vitro* experiments (Table 3). The potential of specific GSK3β inhibitors in treating CKD is increasingly supported by preclinical findings, particularly those involving GSK3β-regulated pathways implicated in renal inflammation, fibrosis, and dysfunction. Among the many tested compounds, lithium, TDZD-8, Tideglusib, and SB216763 are notable for their demonstrated efficacy in reducing kidney injury and fibrosis, making them promising candidates for CKD therapy.

**Table 2:** List of selected GSK3β inhibitors in clinical trials.

Inhibitors	Indications	Clinical trials	Study phase	Target or pathway
Lithium	Cardiac surgery associated AKI	NCT03056248	Phase 4	PI3K/Akt, JAK/STAT3
	Parkinson’s Disease	NCT04273932	Phase 1	Wnt/β-catenin
	Osteosarcoma	NCT01669369	Phase 4	MAPK
	Stroke	NCT01112813	Phase 3	AKT/GSK3β/β-catenin and AKT/FoxO3a/β-catenin
	Amyotrophic lateral sclerosis	NCT00790582	Phase 2	Autophagy
	Low-Grade neuroendocrine tumors	NCT00501540	Phase 2	GSK3β
	Long COVID	NCT06108297	Phase 1	Inflammation
9‐ING‐41	Myelofibrosis	NCT04218071	Phase 2	GSK3β
	Pancreatic cancer	NCT05077800	Phase 2	GSK3β, β-catenin, chemotherapy resistance
	Salivary gland carcinoma	NCT05010629	Phase 2	GSK3β
	Advanced cancers	NCT03678883	Phase 2	GSK3β, PD-1, TIGIT, LAG-3, CXCR3
LY2090314	Metastatic pancreatic cancer	NCT01632306	Phase 2	Cell proliferation
	Acute leukemia	NCT01214603	Phase 2	GSK3α, GSK3β
	Advanced or metastatic cancer	NCT01287520	Phase 1	GSK3α, GSK3β
Tideglusib	Alzheimer´S disease	NCT01350362	Phase 2	Tau
	Alzheimer´S disease	NCT00948259	Phase 2	Tau

AKI, acute kidney injury. COVID, corona virus disease.

**Table 3:** GSK3β inhibitors in animal models.

Inhibitors	Species	Model	Conclusion	Reference
Lithium	C57BL/6 mice	STZ injection	Protects islet β-cells via Nrf2 activation	[192]
	C57BL/6 mice	Ageing	Anti-senescence	[35]
	C57BL/6 mice	Cisplatin or IRI AKI	Promotes recovery from AKI	[176]
	C57BL/6 mice	Cisplatin AKI	Improve autophagy	[177]
	C57BL/6 mice	FA injury	Anti-fibrosis	[91]
TDZD8	C57BL/6 mice	LPS	Anti-inflammation	[67]
	C57BL/6 mice	Doxorubicin injection	Desensitizing MPT	[40]
SB216763	C57BL/6 mice	Doxorubicin injection	Reinforces the Nrf2 antioxidant	[73]
Tideglusib	db/db mice	-	Protects podocytes via Nrf2 activation	[43]

AKI , acute kidney injury. FA, folic acid. IRI, ischemia-reperfusion injury. LPS, lipopolysaccharide. MPT, mitochondria permeability transition. NRF2, NF-E2-related factor 2. STZ, streptozotocin. .

**Figure 5:**
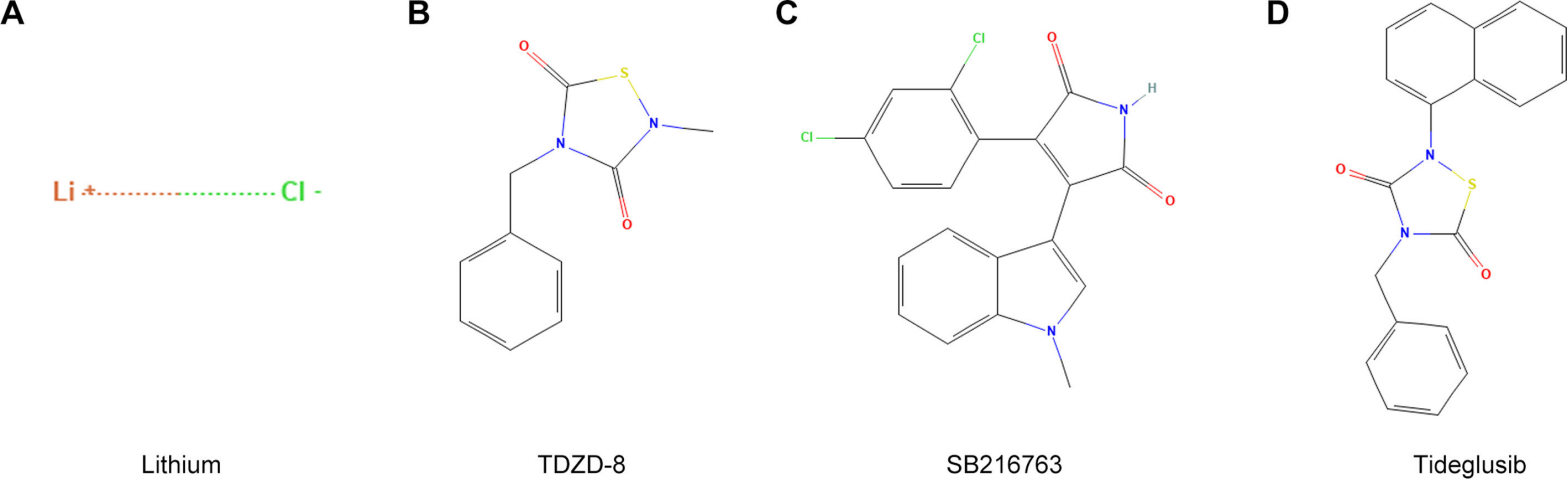
2D chemical structure of the GSK3β inhibitor. (A) 2D chemical structure of lithium chloride. (B) 2D chemical structure of TDZD-8. (C) 2D chemical structure of SB216763. (D) 2D chemical structure of Tideglusib. The 2D chemical structure were download from Pubchem (https://pubchem.ncbi.nlm.nih.gov/).

Lithium, a competitive inhibitor of GSK3 with respect to magnesium (Mg^2+^), has been clinically used for over 50 years as an FDA approved first-line treatment for bipolar affective disorders (Figure 5A), [193]. It is one of the earliest GSK3β inhibitors identified and has been extensively studied for its kidney effects. In kidney disease models, low-dose lithium has shown effectiveness in reducing oxidative stress and fibrosis by stabilizing the Nrf2 antioxidant response and mitigating pro-inflammatory signaling. Interestingly, studies indicate that delayed administration of lithium can also improve outcomes in AKI, reducing the risk of transition to CKD. The renoprotective dose of lithium is much lower than that used for psychiatric diseases, emphasizing the need for precise dosing strategies in CKD to avoid adverse effects while maximizing therapeutic benefit. A randomized, double-blinded, placebo-controlled pilot trial has been conducted on patients undergoing cardiac surgery with cardiopulmonary bypass to assess the effectiveness of short-term low-dose lithium therapy in averting cardiac surgery-associated AKI [191].

TDZD-8, a highly selective non-ATP competitive GSK3β inhibitor, has shown strong efficacy in attenuating fibrosis and inflammation in CKD models (Figure 5B) [40]. By modulating the TGF-β/Smad3 pathway, TDZD-8 reduces fibrotic signaling, making it particularly effective in models of ischemia-reperfusion injury and obstructive nephropathy, where fibrosis is a key pathological feature.

SB216763, a selective ATP-competitive inhibitor of GSK3β, has demonstrated efficacy in reducing podocyte injury and tubular apoptosis in CKD models (Figure 5C). Its ability to activate the Nrf2 antioxidant pathway and reduce NF-κB-driven inflammation makes SB216763 a promising candidate for therapeutic intervention in CKD, supporting both podocyte and tubular cell survival.

Tideglusib, a non-ATP competitive inhibitor of GSK3β, originally developed for neurodegenerative diseases, exhibits renoprotective effects in DKD models by reducing podocyte damage and oxidative stress through GSK3β modulation (Figure 5D) [43]. Tideglusib’s efficacy in protecting glomerular cells suggests it may benefit patients with glomerular forms of CKD, especially those with diabetes-related renal complications.

While these GSK3β inhibitors offer significant promise for CKD treatment, challenges remain, including optimizing dose regimens, enhancing renal selectivity, and evaluating long-term safety. Nonetheless, these inhibitors highlight the potential of GSK3β-targeted therapies to slow CKD progression by addressing core molecular drivers of kidney injury, underscoring the need for continued research into precise and effective therapeutic strategies for CKD.

## Summary

The central role of GSK3β in the progression of CKD is now widely acknowledged. CKD, often stemming from primary conditions such as diabetes and glomerular diseases, progresses through intricate mechanisms driven by chronic inflammation, apoptosis, and fibrosis. GSK3β acts as a key regulatory kinase within multiple signaling pathways, including Wnt/β-catenin, NF-κB, Nrf2, TGFβ-Smad, which together orchestrate critical aspects of kidney damage in CKD. A growing body of evidence provides comprehensive data on GSK3β’s functions across various kidney diseases, such as DKD, PKD, and glomerular diseases, with a focus on its impact on inflammatory responses, fibrotic transformation, and oxidative stress. Evidence from preclinical models indicates that GSK3β inhibition may serve as a therapeutic approach, potentially reducing renal injury and fibrosis while promoting cellular repair. Nonetheless, critical questions remain regarding the long-term effectiveness, specificity, and safety of GSK3β-targeted therapies, with particular concern for potential off-target effects and variability in response across renal cell types. Additionally, debate persists about GSK3β’s mechanistic role in diverse renal pathologies, suggesting further complexity in its function and regulation.

Despite these uncertainties, GSK3β remains a promising therapeutic target with broad implications for CKD management. This review highlights the need for continued research to elucidate the precise functions of GSK3β within renal pathophysiology and to develop effective, targeted therapies that may improve outcomes for patients suffering from CKD and related renal diseases.

## Abbreviations

AKI: acute kidney injury
ARE: antioxidant response element
CKD: chronic kidney disease
CREB: cAMP response element-binding protein
CRMP2: collapsin response mediator protein 2
DKD: diabetic kidney disease
GSK3β: glycogen synthase kinase 3 beta
HG: high glucose
KEAP1: Kelch-like enoyl-CoA hydratase-associated protein 1
LPS: lipopolysaccharide
MAPS: microtubule-associated proteins
MPT: mitochondria permeability transition
NRF2: NF-E2-related factor 2
PKD: Polycystic kidney disease
RISK: reperfusion injury salvage kinase
TECs: tubular epithelial cells
